# PSDX: A Comprehensive Multi-Omics Association Database of *Populus trichocarpa* With a Focus on the Secondary Growth in Response to Stresses

**DOI:** 10.3389/fpls.2021.655565

**Published:** 2021-05-20

**Authors:** Huiyuan Wang, Sheng Liu, Xiufang Dai, Yongkang Yang, Yunjun Luo, Yubang Gao, Xuqing Liu, Wentao Wei, Huihui Wang, Xi Xu, Anireddy S. N. Reddy, Pankaj Jaiswal, Wei Li, Bo Liu, Lianfeng Gu

**Affiliations:** ^1^Basic Forestry and Proteomics Research Center, College of Forestry, Fujian Agriculture and Forestry University, Fuzhou, China; ^2^College of Life Sciences, Fujian Agriculture and Forestry University, Fuzhou, China; ^3^State Key Laboratory of Tree Genetics and Breeding, Northeast Forestry University, Harbin, China; ^4^Department of Biology and Program in Cell and Molecular Biology, Colorado State University, Fort Collins, CO, United States; ^5^Department of Botany and Plant Pathology, Oregon State University, Corvallis, OR, United States; ^6^College of Forestry, Fujian Agriculture and Forestry University, Fuzhou, China

**Keywords:** plant stress, secondary growth, *Populus trichocarpa*, co-expression, alternative splicing, transcriptome

## Abstract

*Populus trichocarpa* (*P. trichocarpa*) is a model tree for the investigation of wood formation. In recent years, researchers have generated a large number of high-throughput sequencing data in *P. trichocarpa*. However, no comprehensive database that provides multi-omics associations for the investigation of secondary growth in response to diverse stresses has been reported. Therefore, we developed a public repository that presents comprehensive measurements of gene expression and post-transcriptional regulation by integrating 144 RNA-Seq, 33 ChIP-seq, and six single-molecule real-time (SMRT) isoform sequencing (Iso-seq) libraries prepared from tissues subjected to different stresses. All the samples from different studies were analyzed to obtain gene expression, co-expression network, and differentially expressed genes (DEG) using unified parameters, which allowed comparison of results from different studies and treatments. In addition to gene expression, we also identified and deposited pre-processed data about alternative splicing (AS), alternative polyadenylation (APA) and alternative transcription initiation (ATI). The post-transcriptional regulation, differential expression, and co-expression network datasets were integrated into a new *P. trichocarpa* Stem Differentiating Xylem (PSDX) database (http://forestry.fafu.edu.cn/db/SDX), which further highlights gene families of RNA-binding proteins and stress-related genes. The PSDX also provides tools for data query, visualization, a genome browser, and the BLAST option for sequence-based query. Much of the data is also available for bulk download. The availability of PSDX contributes to the research related to the secondary growth in response to stresses in *P. trichocarpa*, which will provide new insights that can be useful for the improvement of stress tolerance in woody plants.

## Introduction

Plant growth and development are challenged by a variety of abiotic stresses, such as, cold, drought, high salt and heat (Filichkin et al., 2018). Transcription factors (TF) such as NAC, AP2, and MYB are critical master regulators in stress responses (Lindemose et al., 2013). Identification of transcription factor network and their target genes can help us better understand the transcriptional regulation during secondary growth of woody plants. For example, overexpression of *PtrNAC005* and *PtrNAC100* genes in *Populus trichocarpa* (*P. trichocarpa*) showed a strong enhancement in drought tolerance (Li et al., 2019). Using chromatin immunoprecipitation sequencing (ChIP-Seq) protocol for differentiating stem xylem (Li et al., 2014), transcription factor ARBORKNOX1 (ARK1) has been shown to bind near the transcription start sites of thousands of gene loci implicated in diverse functions (Liu et al., 2015b). In addition to transcription factors, the alteration of histone modification and chromatin modifications can regulate the expression level of genes with stressful environmental changes (Kwon et al., 2009; Kim et al., 2012; Perrella et al., 2013). For example, transcription factor PtrAREB1-2 recruits histone acetyltransferase unit of histone acetylase to enrich H3K9ac and RNA polymerase II specifically at drought-responsive genes *PtrNAC006*, *PtrNAC007*, and *PtrNAC120* containing the ABRE *cis*-element, resulting in increased expression of *PtrNAC* genes (Li et al., 2019).

Splicing factors are the key versatile regulators of alternative splicing (AS) and can cause a significant change in splicing profiles in response to different abiotic stresses (Duque, 2011; Reddy et al., 2013). In plants, over 60% of multi-exon genes undergo AS events (Filichkin et al., 2010, 2018; Zhang et al., 2010; Marquez et al., 2012), and the most prevalent AS type is intron retention (IR) in terrestrial plants. AS is involved in the physiological processes of most plants and can respond to environmental stresses (Staiger and Brown, 2013). Interestingly, stress-responsive AS of transcription factors (TFs) has also been reported (Seo et al., 2013). Previous studies have shown that AS is ubiquitous in *P. trichocarpa* (Baek et al., 2008; Srivastava et al., 2009; Bao et al., 2013; Zhao et al., 2014; Tang et al., 2015; Filichkin et al., 2018) and at least 36% of xylem-expressed genes in *P. trichocarpa* are regulated by AS (Bao et al., 2013). For example, *PtrWND1B*, a key gene regulating secondary cell wall in *P. trichocarpa*, encodes two splice isoforms *(PtrWND1B-s* and *PtrWND1B-l*), which cause antagonism in cell wall thickening during fiber development (Zhao et al., 2014). Moreover, dominant-negative regulators are also reported (Li et al., 2012; Lin et al., 2017).

In addition to AS, alternative transcription initiation (ATI) and alternative polyadenylation (APA) can also produce multiple isoforms from one genetic locus (Xing and Li, 2011). ATI and APA refer to one gene has distinct transcription start sites (TSSs) and transcription termination sites (TTSs). For example, *Lon1* preferentially selects proximal TSS under hypoxic-like conditions and causes mitochondrial dysfunction due to protein misfolding in *Arabidopsis* (Daras et al., 2014). Previous studies have shown that 70, 47.9, and 19.7% genes have more than one poly(A) site in *Arabidopsis*, rice, and moso bamboo, respectively (Wu et al., 2011; Fu et al., 2016; Wang et al., 2017), indicating that APA is a widespread post-transcriptional process. APA events are widely involved in abiotic stress response pathways (Ye et al., 2019; Chakrabarti et al., 2020). Post-transcriptional regulations including AS, and APA are regulated by RNA-binding proteins (RBPs). There are more than 800 RNA binding proteins in *Arabidopsis* (Silverman et al., 2013). The RNA recognition motif (RRM) and the K homology (KH) domain are the most prevalent RNA domains in plants. These RBPs regulate the transcriptome *via* AS, APA, and transcript stability (Bailey-Serres et al., 2020). Thus, it is necessary to investigate the dynamics of these RBPs in response to stress.

The development of high-throughput sequencing technology has enabled us to study the transcriptome and post-transcriptional complexities of plants in different tissues and under different environments (Reddy et al., 2020). Single Molecular Real-Time (SMRT) isoform sequencing technology from PacBio which provides evidence in the form of long sequence reads of the cDNAs (Steijger et al., 2013) has important applications in the identification of post-transcriptional regulation such as complex AS (Zhang et al., 2019; Zhao et al., 2019).

Secondary growth in plants refers to the wood formation due to the activities of the secondary meristem, especially vascular cambium (Mellerowicz et al., 2001; Plomion et al., 2001). *P. trichocarpa* is a model of woody plants with a high-quality reference genome (Tuskan et al., 2006), which largely drives the investigation of secondary xylem development including xylem and phloem differentiation and cell proliferation (Jansson and Douglas, 2007; Kaneda et al., 2010). With increasing number of studies using high-throughput transcriptome sequencing to study secondary growth in poplar, huge amounts of data have been generated and publicly released, which provides extremely valuable information for the investigation of the stem-differentiating xylem (SDX) in response to stress. However, a multi-omics database focused on secondary growth in response to stress has never been reported in *P. trichocarpa*. In order to develop such a resource, we collected the majority publicly available peer-reviewed high throughput data on secondary growth in response to stresses in *P. trichocarpa*, which includes multi-omics data on the TF binding, histone modifications, and transcriptomes. Importantly, we processed and analyzed these datasets with unified parameters and integrated them in a unified *P. trichocarpa* Stem Differentiating Xylem (PSDX) database (http://forestry.fafu.edu.cn/db/SDX). The PSDX database is expected to serve as a valuable resource to the scientific community in understanding the interplay between stress responses and secondary growth in *P. trichocarpa*.

## Materials and Methods

### High Throughput Datasets for Construction of PSDX

**FIGURE 1:**
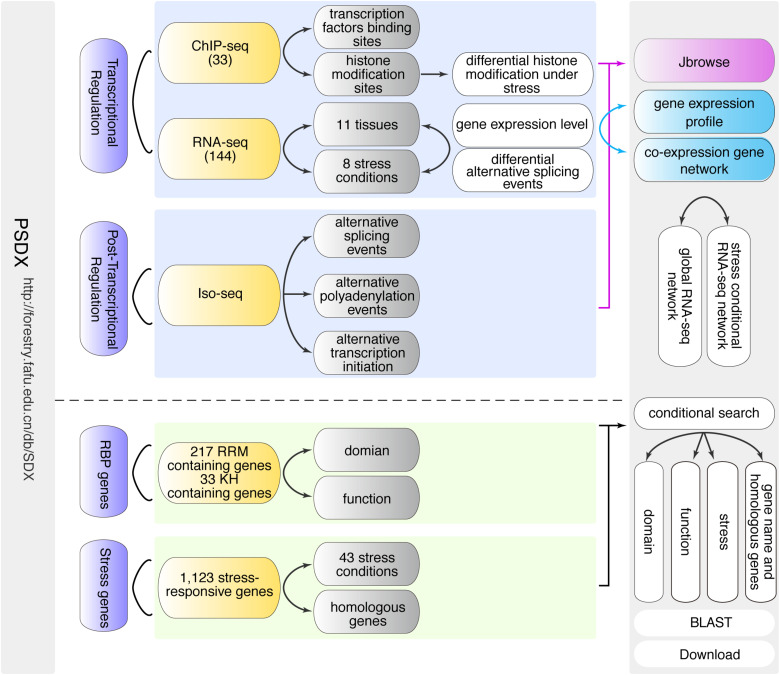
Schematic overview of PSDX database. PSDX consists of two major resources including transcriptional regulation and post-transcriptional regulation under different stress conditions and in different tissues in *P. trichocarpa*. PSDX also includes several other modules: co-expression network, RBP genes, stress-responsive genes, and conditional search.

The *P. trichocarpa* reference genome sequence and annotations (version 3.0) (Tuskan et al., 2006), were obtained from Phytozome (v.12.1.6) (Goodstein et al., 2012). All the raw RNA-Seq, Iso-Seq, and ChIP-Seq datasets were downloaded from the NCBI SRA database through the Aspera tool. Finally, a total of 144 RNA-Seq (Supplementary Table 1) libraries (Lin et al., 2013; Lu et al., 2013; Shi et al., 2017; Filichkin et al., 2018; Li et al., 2019), six Iso-Seq libraries (Filichkin et al., 2018) and 33 ChIP-Seq (Supplementary Table 1) libraries (Liu et al., 2014, 2015a,b; Li et al., 2019) were included in this study (Figure 1). Besides, the RNA-binding proteins containing RRM and KH domains from *Arabidopsis* were retrieved from a previous study (Lorkovic and Barta, 2002) to search for homologs in *P. trichocarpa*.

#### ChIP-Seq Data Analysis

**FIGURE 2:**
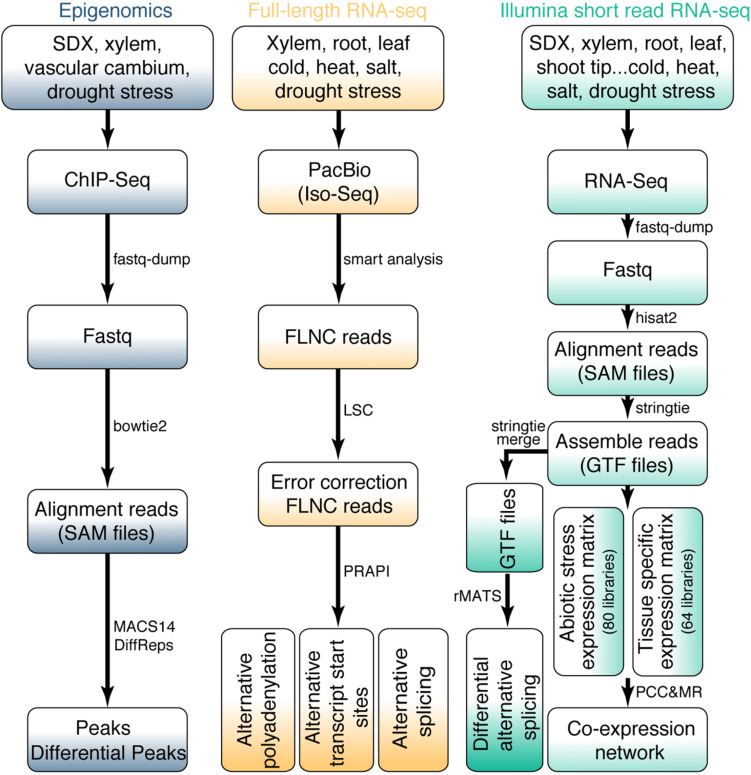
Bioinformatics workflow for omics data. The upper part of the figure shows the data source for RNA-seq and epigenomics. The bioinformatics pipeline for all data processing is shown in the middle and bottom of the figure.

For the identification of TF peaks or histone modification sites based on ChIP-Seq data, the sequence reads were aligned to *P. trichocarpa* reference genome using bowtie2 v.2.2.1 (Langmead and Salzberg, 2012) with “-k 2 –no-unal –no-hd –no-sq” parameters. MACS14 was used for peak calling (Zhang et al., 2008). DiffReps (Shen et al., 2013) was used to analyze differential peaks in ChIP-seq signals with “–pval 0.05” parameters (Figure 2).

#### RNA-Seq Data Analysis

In total, 144 RNA-Seq libraries were subjected to transcriptome analysis to calculate gene expression levels and identify AS events. The raw FASTQ files were extracted from the SRA files using fastq-dump v.2.5.7 (Leinonen et al., 2011). Then ht2-filter v1.92.1 of the sequence filter package HTQC (Yang et al., 2013) was used to filter the low-quality bases from single or pair-end reads. The filtered reads were aligned to the *P. trichocarpa* reference genome with hisat2 v.2.0.3-beta (Kim et al., 2015). Only the unique reads were kept. Samtools v.0.1.7 (Li et al., 2009) was used to convert the uniquely SAM files to sorted bam files. Gene expression levels were normalized to FPKM (Fragments Per Kilobase of transcript per Million mapped) for paired-end libraries and RPKM (Reads Per Kilobase of transcript per Million mapped reads) for single-end libraries, respectively. The edgeR package was used for differential gene expression analysis (Robinson et al., 2010). The *P* values less than 0.05 and | Fold Change| greater than 1.5 were considered as statistically significant. In order to identify different AS events, the transcript data was assembled and merged using StringTie v.1.3.3b (Pertea et al., 2015). The obtained BAM file and merged GTF file were used to identify AS events using rMATS v.3.2.2.beta (Shen et al., 2014) with following parameter: -a 8 -c 0.0001 -analysis U –keepTemp to identify differential AS events with false discovery rate (FDR) < 0.05 (Figure 2). AS events was visualized by rmats2sashimiplot^1^.

#### Iso-Seq Data Analysis

For the analysis of Iso-Seq data, we obtained high-quality reads of insert (ROI) by ConsensusTools script of SMRT Analysis v.2.3.0 software package from Pacific Biosciences. Then we used pbtranscript.py of SMRT Analysis v.2.3.0 software package to obtain full-length non-chimeric (FLNC) reads. For a small part of the chimeric ROI, they were marked as complete ROI by judging the 5′ primer, 3′ primer, presence of poly(A) and corresponding positional relationship. LSC 1.alpha (Au et al., 2012) was used to correct Iso-Seq. Finally, these corrected Iso-Seq reads were further used as an input file to identify AS events using PRAPI (Gao et al., 2018) with default parameters to identify the post-transcriptional regulation events including ATI, AS, and APA (Figure 2).

### Identification of RNA-Binding Protein Coding Genes in *Populus trichocarpa*

The RBPs genes in *Arabidopsis* have previously been reported (Lorkovic and Barta, 2002). Firstly, the protein sequence containing the RRM and KH domains was extracted from *Arabidopsis* (TAIR10) gene loci. Then, hmmbuild (Potter et al., 2018) was used to build HMMER matrices with all RBP candidate genes in *Arabidopsis.* Next, homologous RBP candidate genes in *P. trichocarpa* were identified by hmmsearch tool with -E 10^–5^. All conserved domains of RRM and KH from RBP candidate genes were confirmed by NCBI-CDD.

### Identification of Stress Response Genes in *Populus trichocarpa*

In this study, we collected stress tolerance or stress response genes from several public databases and literature sources. To do this, we started by collecting 617 *Arabidopsis* stress tolerance or stress response genes from ASRGDB (Borkotoky et al., 2013) and 106 drought stress response genes in *Arabidopsis* from DroughtDB (Alter et al., 2015). Additionally, we performed a comprehensive review and biocuration of published literature (Villar Salvador, 2013; Hossain et al., 2016; Vats, 2018; Singh et al., 2019). In total, we collected a list of 766 unique *Arabidopsis* stress response genes. To identify putative stress response genes in *P. trichocarpa*, we used blastall (-p blastp, -e 1e-5) with the amino acid sequence of 766 *Arabidopsis* genes as the query. A set of 1,123 *P. trichocarpa* gene with amino acid sequence similarity of more than 50% were identified and retained as candidate stress response genes (Supplementary Table 2).

### Co-expression Networks Analysis

For the measure of gene co-expression network, we compared the cut-off values of Pearson Correlation Coefficient (PCC) with mutual rank (MR) score (Obayashi and Kinoshita, 2009). The MR values were calculated as described previously (You et al., 2016). According to the methods in previous study (Obayashi and Kinoshita, 2009; You et al., 2016; Tian et al., 2018), we extracted 176 genes with the number of GO terms ranging from 4 to 20 using Receiver Operating Characteristic (ROC) curve and Area under the ROC Curve (AUC) value to evaluate the constructed co-expression network as a specific binary classifier. Based on the co-expression networks with thresholds of PCC > 0.54, PCC > 0.64, PCC > 0.74, PCC > 0.84, MR top3 + MR ≤ 30 or MR top3 + MR ≤ 100, we inferred the true or false determination for GO from pair-wise genes. We used co-expression gene pairs (Potri.008G069600 and Potri.004G162400) from threshold of MR top3 + MR ≤ 30 as example. Potri.008G069600 included four GO terms (GO:0003674, GO:0071704, GO:0003824, and GO:0006629). Potri.004G162400 included five GO terms (GO:0003674, GO:0003824, GO:0005975, GO:0006006, and GO:0004332). Using Potri.004G162400 as prediction, Potri.008G069600 shown five predicted GO terms including GO:0003674, GO:0003824, GO:0005975, GO:0006006, and GO:0004332. Comparing with the original GO terms of Potri.008G069600 (GO:0003674, GO:0071704, GO:0003824, and GO:0006629), only GO:0003674 and GO:0003824 were true (marked as 1). GO:0005975, GO:0006006, and GO:0004332 were false (marked as 0) (Supplementary Table 3). Finally, we selected different thresholds to calculate TPR (true positive rate, TPR) and FPR (false positive rate, FPR). ROC curves and AUC were generated using sklearn plugin. The expression profile and co-expression module was displayed using script from ALCOdb (Aoki et al., 2016).

### Database Construction

*Populus trichocarpa* Stem Differentiating Xylem portal consists of a front-end user interface and a back-end database. The database mainly includes information such as FPKM/RPKM, AS events, APA, ATI, RBP candidate genes, and gene functions, which are presented to the user through the search function module (Figure 1). The search function was divided into simple fuzzy (approximate) string searching, single condition or multiple condition joint search, and BLAST search. Both the ‘‘Home page’’ and the ‘‘Search page’’ contained the entry of a simple fuzzy string search enabled by the PHP. The user can input any keyword by fuzzy string searching to query data in MySQL database, and the matching results will be returned to the user. The results data table is displayed using the Bootstrap extension. BLAST search allows the users to perform a sequence-based query. The best matches connect to the gene pages. For the BLAST graphical user interface, we applied a Sequence Server^2^ with a modern graphical user interface to set up BLAST+ server (Priyann et al., 2019). Multiple text downloads make it quick and easy to customize the dataset. The JBrowse (Buels et al., 2016) interface is provided to query and visualize the data aligned to the reference genome.

## Results and Discussion

### Database Overview and Essential Modules of PSDX

In order to facilitate researchers to access and mine high-throughput sequencing information related to secondary growth upon stress response in *P. trichocarpa*, we constructed an easy-to-use and fully functional web database to query, download, visualize and integrate multi-omics data using a unified portal. The search-query function is the core of the PSDX database, which includes two different built-in search capabilities, including keyword search and BLAST-search (Figure 1). When users provide keywords such as gene names, function descriptions, or GO terms in the search box, which will present a drop-down list of search suggestions to auto-complete the keywords (Figure 1). After the user submits the keyword, the query function will return a comprehensive data sheet containing pre-computed information based on high throughput sequencing and presents it with a high-resolution figure, and JBrowse genome browser hyperlink.

The gene page includes visualization of the gene structure, gene expression from RNA-Seq, post-transcriptional events from RNA-Seq and Iso-Seq, and other feature information such as functional annotation. The genome browser includes 206 tracks which include the reference genome, gene structure (GFF), and bigwig files for all ChIP-Seq, RNA-Seq and Iso-seq libraries. Users can also upload their local data in standard formats like the BAM, BED, GFF, GTF, VCF, etc., to compare alongside the existing data.

For researchers, interested in profiling sequence-based queries, PSDX’s BLAST function module is a preferred tool. Users can paste their own sequence in FASTA format into the text box or drag the FASTA file directly into the text box. Then the system will automatically recognize whether it is a protein sequence or a nucleic acid sequence. After the selection of the appropriate alignment database, the BLAST submit button will be activated, which dramatically reduces the user’s burden. After performing the BLAST comparison, the web browser displays a comparison result report in HTML, XML, or CSV format, which are summarized with the details of each matching alignment and overview graph including the alignment strength and position of each hit. After clicking on the gene name in the BLAST hit results, users can also hyperlink to the detailed page of the PSDX database to view all the information about the gene.

For the export of data, PSDX provides downloads in diverse formats, including CSV, MS-Excel, and TXT. The user can perform a secondary screening of the table in real-time by entering keywords to obtain more accurate results by filter box for the table, which is located at the top right of the data table. Finally, PSDX has created online documentation, which provides a quick start guide on searching, mining, browsing, downloading, and other more comprehensive Supplementary Material.

In summary, we integrated transcription factor peaks, gene expression, co-expression network, post-transcriptional regulation information, stress response genes, and RBP candidate genes into one unified database. In the following section, we presented a detailed description of the above six modules.

### TF and Differential Histone Modification in Response to Abiotic Stress

To generate a comprehensive chromatin state map of *P. trichocarpa*, we collected the majority of published ChIP-seq libraries, which included peaks of several transcription factors such as ARK1, ARK2, BLR, PCN, and PRE. In total, 15,351 genes with 47,552 TF peaks were identified and integrated in the PSDX database (Supplementary Table 4). In addition to TF, H3K9ac modifications are known for their role in drought response in *Arabidopsis* (Kim et al., 2012) and *P. trichocarpa* (Li et al., 2019). Histone acetylation showed alteration in the drought stress-responsive genes under drought stress in *P. trichocarpa* (Li et al., 2019). Thus, we also identified 37,112, 41,613, and 35,703 peaks from different H3K9ac ChIP-seq data (Supplementary Figure 1 circle 2, 5, and 8). In total, the PSDX database contains 8,359 and 9,360 differential H3K9ac modifications (Supplementary Table 4) for 5- and 7-day without watering (drought-like conditions), respectively. The level of H3K9ac also changed in differentially expressed genes under drought stress or drought-responsive genes (Supplementary Figure 1 circle 3, 4, 6, 7, and 9), which could be visualized at PSDX.

### Differential Gene Expression Analysis in Response to Abiotic Stress

In this study, we calculated gene expression in diverse tissues including root, callus, leaf, stem, stem xylem, and shoot tip. Furthermore, we also investigated gene expression patterns under cold, salt, heat, and drought stress conditions. A principal component analysis identified tissues (Supplementary Figure 2A) rather than stresses (Supplementary Figure 2B), as the main factor driving gene expression, which was consistent with previous research (Appels et al., 2018). In addition, we also conducted Principal Component Analysis and found that the samples can be separated by tissues and treatments (Supplementary Figure 3). Next, to identify differentially expressed genes involved in secondary growth, we compared gene expression levels of secondary xylem with that of other tissues. In total, we show that over 21,442 genes are differentially expressed in secondary xylem compared with other tissues. To further investigate which genes are affected in xylem under different stress conditions, we performed differential expression analysis between non-stress and stress conditions in the xylem. In total, we found 19,872 differentially expressed genes in response to different stresses in xylem, which included previously reported Potri.002G081000 (*PtrNAC006*) (Li et al., 2019). Overexpression of Potri.002G081000 (*PtrNAC006*) could significantly improve drought-adaptive capabilities through an increase in the number of xylem vessels (Li et al., 2019). In the search pages of our PDSX, users can use Potri.002G081000 (*PtrNAC006*) as a search condition to get the highly expressed pattern of Potri.002G081000 (*PtrNAC006*) under drought and other stresses in secondary xylem. The user also can get all other specifically expressed genes of secondary xylem in response to different stress.

### Stress-Specific Co-expression Networks

We followed previous method (Obayashi and Kinoshita, 2009; You et al., 2016; Tian et al., 2018). In brief, PCC between each pair of genes (A and B) was calculated. The mutual rank (MR) is a refinement of geometric mean of the ranked PCC: MR(AB) = $\sqrt{Rank\left(AB\right)\times Rank\left(BA\right)}$. AB represents the order of gene A in gene B’s PCC list, whereas BA represents the order of gene B in gene A’s PCC list. The highest 5% PCC value of all positive co-expression gene pairs were 0.54 under stress samples (Supplementary Figure 4). We selected four PCC thresholds (PCC > 0.54, 0.64, 0.74, and 0.84). Beyond that, we selected two MR thresholds including MRtop3+MR ≤ 30 or MRtop3+MR ≤ 100. Then, we generate receiver operating characteristic (ROC) curves to compare the performance of different classifiers. The detail steps of how to evaluate the superiority of different classifiers is described in methods. As a result, the AUC of the co-expression network under PCC > 0.74 as cut-off was the best (Supplementary Figure 5A). In addition, co-expression networks with thresholds of MR top3+MR ≤ 30 and MR ≤ 100 were tested and the network with MR top3+MR ≤ 30 was the best in all cut-off (Supplementary Figure 5B). Thus, we used MR top3+MR ≤ 30 as cut-off values for co-expression networks in this study.

### Differential Alternative Splicing in Response to Abiotic Stress

**FIGURE 3:**
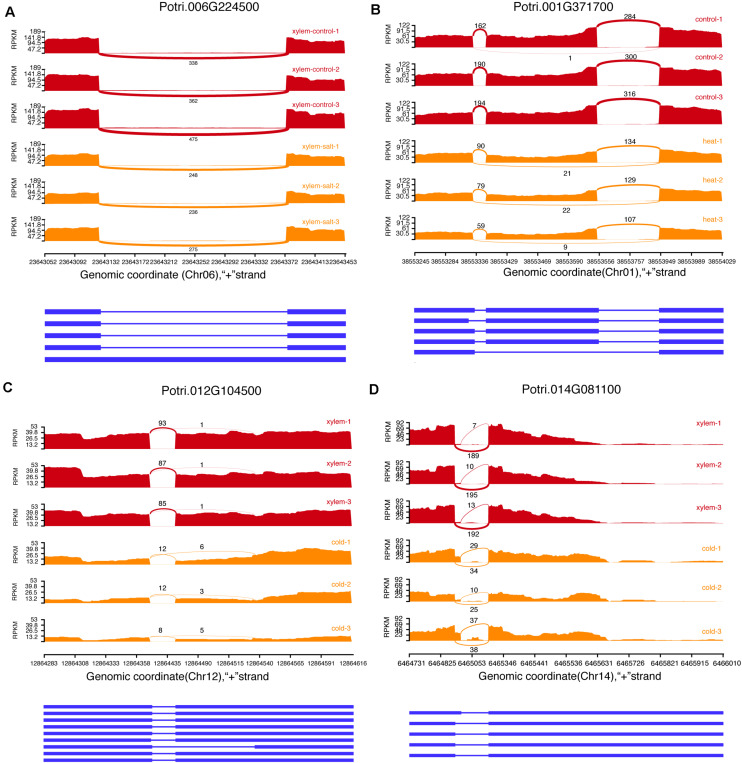
Examples of four alternative splicing events. **(A)** Intron retention (IR). **(B)** Exon skipping (ES). **(C)** Alternative acceptor sites (AltA). **(D)** Alternative donor sites (AltD).

We identified 78,526 AS events in 16,545 genes from 144 transcriptome datasets sampled from different stress and tissues. The most common AS types included Exon skipping (ES), Intron Retention (IR), Alternative acceptor sites (AltA), and Alternative donor sites (AltD), respectively (Figure 3). The AS events include 28,773 Intron Retention (IR), 13,348 Exon skipping (ES), 24,087 Alternative acceptor sites (AltA), and 12,318 Alternative donor sites (AltD), respectively (Supplementary Table 5). Especially, we analyzed the difference in AS events between secondary xylem and other tissues under normal conditions, which revealed 2,178 differential AS events in xylem with secondary growth (Supplementary Table 6). To further investigate how AS events affect xylem under different stress conditions, we analyzed differential AS between non-stress control and stress conditions (cold, salt, heat, and drought). In total, 6,559 genes presented 14,003 differential AS events which included 275 stress response genes. For example, Potri.001G448400 (*PtrWND1B*), a key gene regulating secondary cell wall thickening in *P. trichocarpa*, is shown to occur AS in secondary xylem fiber cells (Zhao et al., 2014). In the search pages of PDSX, users can use Potri.001G448400 as a search condition to get the differential AS pattern of this gene under different stresses in secondary xylem. Apart from differential AS in xylem under stress treatment, we also identified 24,103 differentially expressed AS events under other comparisons, which could be searched and visualized at PSDX.

### Post-transcriptional Regulation Based on Iso-Seq

Single-molecule real-time (SMRT) Isoform Sequencing (Iso-Seq) presents a great advantage in the identification of post-transcriptional regulation based on full-length splicing isoforms (Gao et al., 2019; Zhang et al., 2019; Zhao et al., 2019). Using Iso-Seq data we identified a total of 58,000 AS events in 9,199 genes which included 22,882 IR, 4,175 ES, 17,031 AltA, and 13,912 AltD, respectively (Supplementary Table 7). Among them, 9,490 AS events were identified in both RNA-seq data and PacBio Iso-seq data (Supplementary Table 8). However, RNA-seq libraries presented more AS events which did not detected by PacBio since the 144 RNA-seq libraries contained more tissues and treatment conditions. Subsequently, we merged the gene annotation of RNA-seq and Iso-seq by stringTie and compared merge GTF file with reference annotation using gffcompare (Pertea and Pertea, 2020). The merged GTF file covered all the annotated transcripts. In addition, we obtained 15,720 transcripts within intergenic regions, which did not cover in original annotated transcripts. These new loci can be obtained from download module of PSDX. In addition to AS, we applied PacBio sequencing to identify genome-wide APA and ATI events in *P. trichocarp*a. In total, we identified 165,455 polyadenylation sites from 26,589 genes, of which 21,455 genes had more than one polyadenylation site. Among them, there are 18,637 genes in the same tissue in the control sample. Furthermore, 18,021 genes showed APA events under stress conditions of which 3,590 genes are specifically induced by stress (Supplementary Figure 6A). Meanwhile, a comparison of APA genes between different stress and control tissues revealed that APA genes are changed under different stresses (Supplementary Figures 6B–D). Further analysis revealed 14,922 genes including 39,606 ATI events (Supplementary Figure 6E), which also presented a dynamic change in different tissues and stresses (Supplementary Figures 6F–H). All these AS/APA/ATI could be searched and visualized from PSDX.

### Compendium of RNA-Binding Genes in Response to Abiotic Stress

**FIGURE 4:**
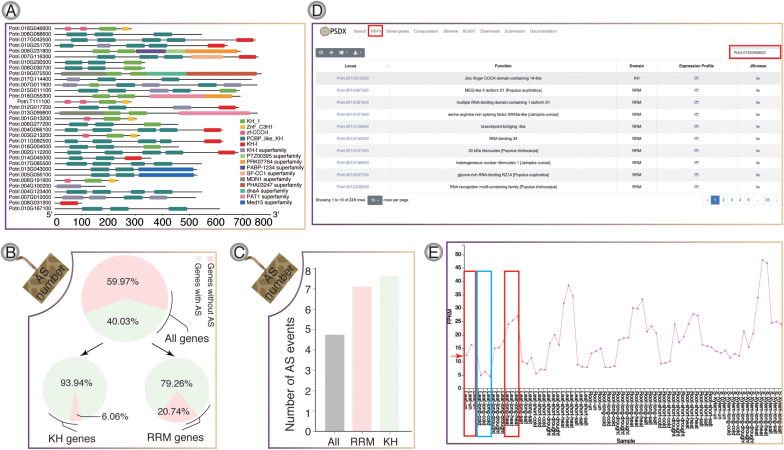
RNA-binding protein genes in *P. trichocarpa*. **(A)** KH-domain structure diagrams. **(B)** Venn diagram shows the percentage of AS in all genes, KH-containing genes, and RRM-containing genes, respectively. **(C)** The average number of AS events for RRM and KH domain genes, respectively. **(D)** The module of RNA binding protein. **(E)** Splicing factor Potri.015G004600 (*PtrSR34A*) was selected as one case, which showed up-regulation upon heat stress (red box) and down-regulation upon cold stress (blue box).

RNA-binding proteins genes in *P. trichocarpa* were predicted using hmmbuild (Potter et al., 2018) and hmmsearch. In total, we identified a total of 217 RRM-containing genes (Supplementary Figure 7) and 33 KH-containing genes (Figure 4A), respectively. In *P. trichocarpa*, 40% of the genes had AS events, while the percentage of AS events for RBP containing the KH and RBP domain is about 94 and 79%, respectively. It was obvious that RBPs showed a higher percentage of AS. The high frequency of AS events in RBPs is consistent with previous studies, which showed that splicing factors are regulated by AS of their own mRNAs (Zhang et al., 2017). RRM and KH domain-containing genes have more splicing isoforms (Figures 4B,C). All the stress-induced information for these RBPs could be searched in RBPs pages. Taking Potri.015G004600 (*PtrSR34A*) as an example (Figure 4D), *PtrSR34A* showed differential regulation in response to cold and heat stress, respectively (Figure 4E). These dynamics change of these RBP in response to stress could play important roles in widespread AS change.

### Compendium of Stress Response Genes

**FIGURE 5:**
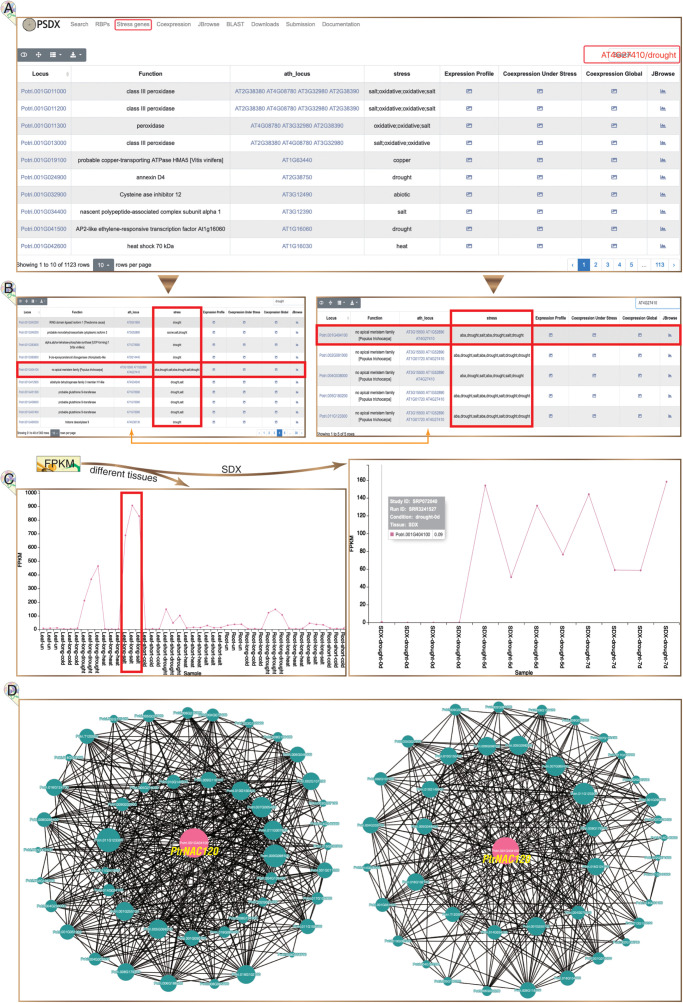
Stress response genes in *P. trichocarpa*. **(A)** The module of stress response genes. **(B)** Following searches, all *P. trichocarpa* genes homologous to AT4G27410 (left panel) and all drought stress-responsive genes (right panel) are returned from a query. **(C)** Potri.001G404100 (*PtrNAC120*) is up-regulated upon drought and salt stress, especially significantly up-regulated under salt stress in leaves (left panel). **(D)** Stress-responsive genes co-expression network is presented under stress treatment libraries (left panel) and all RNA-seq libraries (right panel), respectively.

In total, 1,123 highly reliable stress response genes were imported into PSDX from module of “Stress genes” (Figure 5A). Interestingly, we found that 76% (867) stress response genes have APA events, which suggested that stress response genes are regulated by APA. This module of “Stress genes” presents all key co-expression profiles under different stresses. The keyword of gene name of *P. trichocarpa* or the homologous gene in *Arabidopsis* can be inputted in the search box. Using drought marker gene Potri.001G404100 (*PtrNAC120*) as an example, the expression profile, co-expression profile, and response in multiple stress can be returned in the result page (Figure 5B). *PtrNAC120* showed high up-regulation not only in drought stress but also in salt stress (Figure 5C), which indicated that *PtrNAC120* may also play vital roles in salt response. Co-expression profiles shown that, compared to global network, stress-specific co-expression networks had strong evidence for Potri.011G123300 (*PtrNAC118*) co-expressed with *PtrNAC120* (Figure 5D), which was reported previously (Li et al., 2019). In addition to the locus name of *P. trichocarpa*, this page is also searchable by *Arabidopsis* homologs locus name.

### Case Study of Database Usage

**FIGURE 6:**
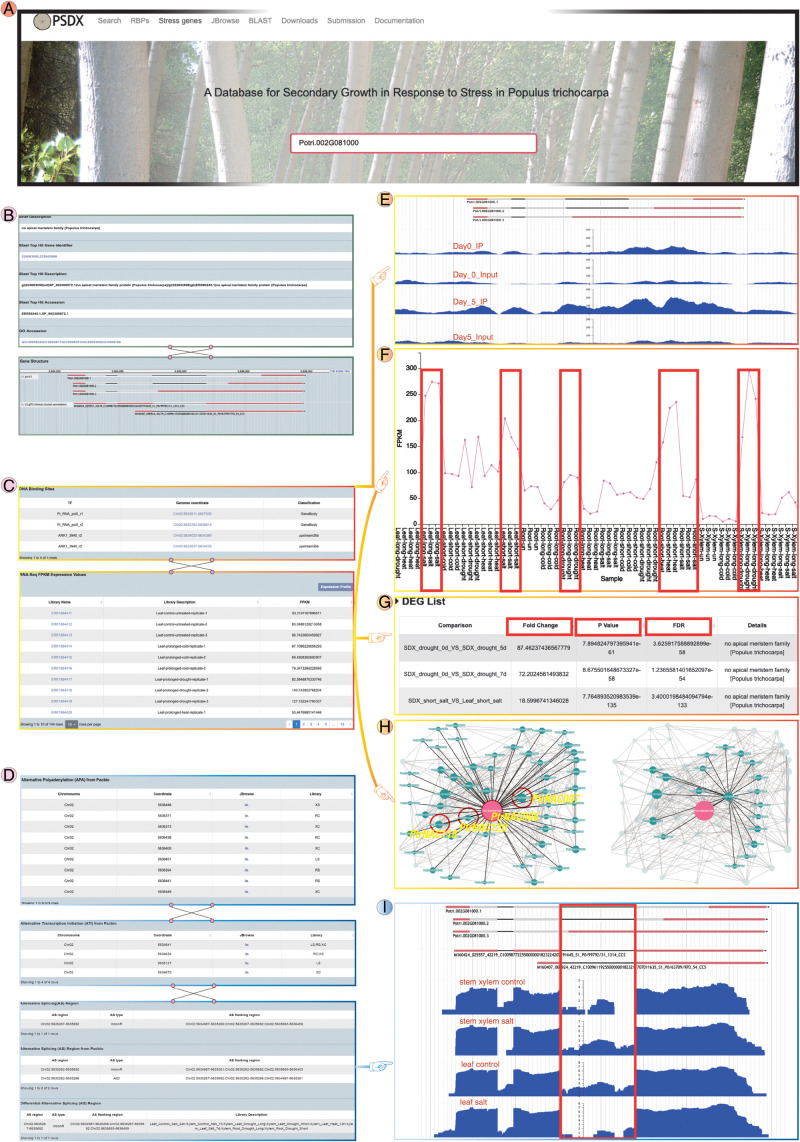
Potri.002G081000 (*PtrNAC006*) was selected as an example to show multi-dimensional omics levels in PSDX. **(A)** PSDX main search interface with Potri.002G081000 (*PtrNAC006*) as an example. **(B)** A brief introduction and gene structure of *PtrNAC006*. **(C)** Transcriptional regulation of *PtrNAC006*. The top panel is the DNA peaks of *PtrNAC006*. All the FPKM values are provided in the bottom panel. **(D)** Post-transcriptional regulation of *PtrNAC006*, which including APA sites, ATI, AS events from short-read RNA-seq and PacBio Iso-seq, and differential AS events. **(E)** Wiggle plot shows increased H3K9ac levels under drought stress. **(F)** *PtrNAC006* is significantly up-regulated under drought and salt stress, respectively. **(G)** Differential expression of *PtrNAC006* in different organs and stresses, users can re-rank the DEG with fold change, *P*-value, and FDR. **(H)** Co-expression network of *PtrNAC006*, which connected with other drought-responsive genes Potri.007G099400 (*PtrNAC007*), Potri.011G123300 (*PtrNAC118*), and Potri.001G404100 (*PtrNac120*). **(I)** The second intron of *PtrNAC006* tends to be included (intron retention) under salt stress in stem xylem. However, the intron tends to be fully spliced under salt stress in the leaf.

Users can select detailed information according to their own interests. PSDX exhibited differential expression in various tissues under stress and normal conditions. For example, Potri.002G081000 (*PtrNAC006*) showed higher levels in all stress conditions, especially in the drought stress. Taking the query results of drought marker gene *PtrNAC006* as an example (Figure 6A). The “main information” page shows some basic information of *PtrNAC006* including the function, gene structure, GO annotation, and sequence (Figure 6B). On this page, PSDX also shows transcriptional regulation and post-transcriptional regulation of *PtrNAC006*. For transcriptional regulation of *PtrNAC006*, transcription factor peaks and FPKM in all tissues and stresses can be found on this page (Figure 6C). For post-transcriptional regulation, the profile of APA, AS, and ATI can be found in “main information” (Figure 6D). The user also can visualize the enrichment of peaks and FPKM values of *PtrNAC006* among different tissues and stresses (Figures 6E,F). From the return page, *PtrNAC006* is highly expressed in drought stress, consistent with previous studies (Li et al., 2019; Figure 6F). In the “DEG list” module user can filter and sort DEG genes based on fold change, *P* values and FDR (Figure 6G). In the “DAS list” module user can also find the DAS events (Figure 6G). In the “Co-Expression” page user can obtain other genes that are closely related to the queried gene in expression patterns. *PtrNAC006* is highly co-expressed with Potri.007G099400 (*PtrNAC007*), Potri.011G123300 (*PtrNAC11*8) and Potri.001G404100 (*PtrNAC120*) (Figure 6H), which was reported previously (Li et al., 2019). For the post-transcriptional regulation, the second intron of *PtrNAC006* is preferentially retained in stem under salt stress. However, the opposite trend was found in leaf and this intron is spliced fully in leaf under salt stress (Figure 6I). It will be interesting to investigate the function of these differential splicing isoforms in the future since they show an obvious change in response to stress.

## Conclusion and Future Directions

*Populus* is a commercial plantation species due to cellulose and lignin in secondary walls in papermaking (Jansson and Douglas, 2007). However, the mechanisms regulating development in response to different stresses are not yet clear in post-transcriptional level. In this study, we integrated 144 RNA-Seq libraries and 6 Iso-Seq libraries to get information about the normalized gene expression and differential expression analysis between non-stress and stress conditions. Additionally, we also used 33 ChIP-seq libraries from different stresses and tissue to reveal different levels of regulation, which included histone modification sites and TF peaks. Thus, PSDX provided a platform for an integrated analysis of multi-omics data and especially focuses on multi-omics data for wood development in response to stress. With available modern biotechnologies (Lin et al., 2006), PSDX will provide a preliminary resource for characteristics of secondary xylem development using transgenic lines with modified wood-related genes to generate superior wood quality in future.

For the post-transcriptional regulation, 40,284 differential AS events in response to stress were specifically identified. Apart from AS, we identified 21,455 genes with more than one polyadenylation site. Post-transcriptional results were also integrated into the PSDX database, which provides a variety of search methods to query the gene expression and post-transcriptional regulation information of *P. trichocarpa*.

*Populus trichocarpa* Stem Differentiating Xylem supports the export of search results and download of all original datasets. PSDX also offers a powerful visualization tool and modern BLAST sequence alignment tool, both of which are not only common tools, but also tightly integrated with the data carried by PSDX. For example, a matching gene obtained by BLAST search can be linked to a page of detailed information such as FPKM and AS in different tissues and in response to different stress. With more research on the growth of trees, new high-throughput data from Illumina, PacBio, and Nanopore platforms PSDX data will be processed and released when available.

## Data Availability Statement

The original contributions presented in the study are included in the article/Supplementary Material, further inquiries can be directed to the corresponding author/s.

## Author Contributions

LG, WL, and BL conceived and designed the study. HyW, SL, XD, YY, YL, YG, XL, WW, HhW, and XX collected data and conducted analyses. HyW, SL, AR, PJ, and LG contributed to the interpretation of results and drafting the manuscript. All authors read and approved the final manuscript.

## Conflict of Interest

The authors declare that the research was conducted in the absence of any commercial or financial relationships that could be construed as a potential conflict of interest.

## Footnotes

1 https://github.com/Xinglab/rmats2sashimiplot/

2 http://sequenceserver.com

## Supplementary Material

Supplementary Figure 1 Genome-wide distribution of H3K9ac enrichment in *Populus trichocarpa* under drought stress, which is presented in nine circles from 1 (innermost circle) to 9 (outermost circle). The inner-circle 1 represents gene density. Circle 2 shows H3K9ac enrichment distribution after 7 days drought. The color scale ranges from red (high enrichment) to blue (low enrichment). Circle 3 presents differential H3K9ac levels distribution under 7 days drought. The orange dot is increased both H3K9ac level and gene upregulation, the green dot represents a decrease in both H3K9ac level and gene downregulation, the blue dot shows opposite regulation of H3K9ac level and gene regulation, and the yellow dot shows only differential H3K9ac levels. Circle 4 presents differential genes after 7 days of drought (the orange dot shows increased H3K9ac level and gene upregulation, the green dot shows decreased in both H3K9ac level and gene downregulation, the blue dot shows opposite regulation of H3K9ac level and gene regulation, the yellow dot shows only differential gene regulation). Circle 5 presents H3K9ac enrichment distribution after 5 days of drought. Circle 6 shows differential H3K9ac levels distribution after 5 days of drought. Circle 7 presents differentially expressed genes after 5 days of drought. Circle 8 presents H3K9ac enrichment distribution after 0 days drought. Circle 9 presents chromosomes of *P. trichocarpa* and the red line in the track represents drought-responsive genes, which showed that H3K9ac modifications are enriched in drought-responsive genes.

Supplementary Figure 2 Principal component (PC) analysis plots for different tissues and stress treatments. **(A)** Principal component (PC) analysis plots for different tissues. Different shape means different tissues. “Other” group represent protoplasts with miR397, SND1 or GFP overexpression. “SDX” group represent Stem Differentiating Xylem. **(B)** Principal component (PC) analysis plots for different stress treatments. Different shape means different treatments. “Other” group represent protoplasts with miR397, SND1, or GFP overexpression.

Supplementary Figure 3 Principal component (PC) analysis plots for different tissues and datasets. Different shape means different datasets. Different color means different tissue. “SDX” group represent Stem Differentiating Xylem.

Supplementary Figure 4 The distribution of the Pearson Correlation Coefficient of stress-responsive genes under stress.

Supplementary Figure 5 MR values filtering for stress co-expression network. **(A)** Assessment of PCC co-expression network. **(B)** Assessment of MR co-expression network.

Supplementary Figure 6 The profile of alternative polyadenylation and alternative transcription initiation. **(A)** All APA genes in control and stress condition. **(B)** APA genes under stress and control in leaf. **(C)** APA genes under stress and control in root. **(D)** APA genes under stress and control in stem xylem. **(E)** All ATI genes in control and stress condition. **(F–H)** ATI genes in leaf, root and stem xylem compare control and stress.

Supplementary Figure 7 Domain structure diagrams for RRM-domain protein.

Supplementary Table 1 Sequencing data used in this study.

Supplementary Table 2 *P. trichocarpa* stress response genes.

Supplementary Table 3 Values for an example of ROC analysis.

Supplementary Table 4 ChIP-seq peaks. Sheet 1 are peaks from transcription factors of ARK1, ARK2, BLR, PCN, and PRE. Sheet 2 are 9,360 differential H3K9ac modifications for 7-day without watering. Sheet 3 are 8,359 differential H3K9ac modifications for 5-day without watering.

Supplementary Table 5 Alternative splicing events from 144 RNA-seq libraries. The first column is the locus name, and the second column is AS events from this gene. Each AS event contains the information of location and type.

Supplementary Table 6 Differential alternative splicing in stem-differentiating xylem. The first column is the locus name, and the second column is the location information of the AS.

Supplementary Table 7 Alternative splicing events from Iso-seq. The first column is the locus name, and the second column is all AS events of this gene.

Supplementary Table 8 Overlapped alternative splicing events between RNA-seq and Iso-seq.
