# Supplementary figures and images for: PSDX: A Comprehensive Multi-Omics Association Database of Populus trichocarpa With a Focus on the Secondary Growth in Response to Stresses
Source: Front Plant Sci. 2021 May 20;12:655565. doi: 10.3389/fpls.2021.655565 (PMC8195342; doi:10.3389/fpls.2021.655565)

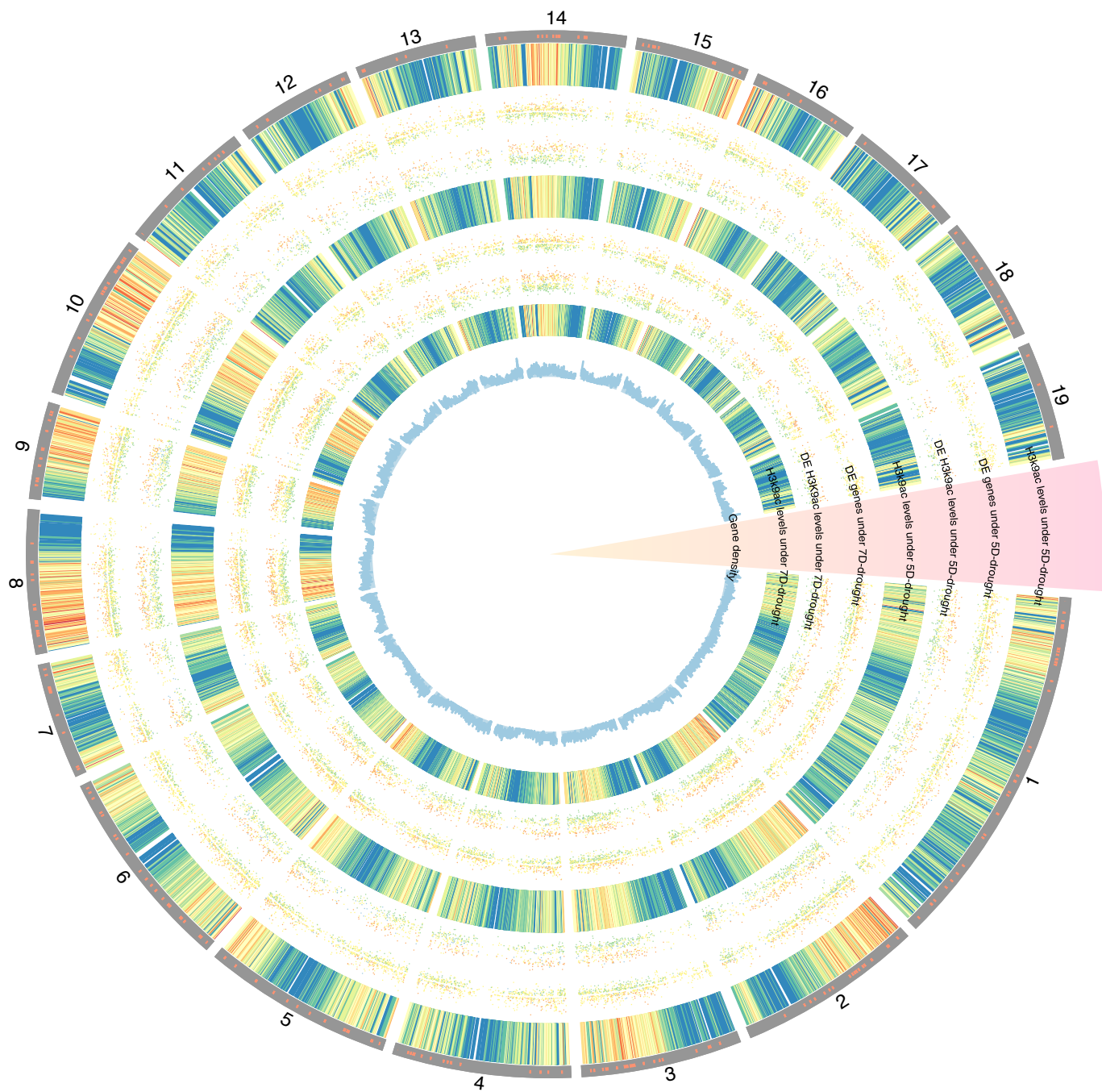

Supplement: Supplementary Figure 1 — Genome-wide distribution of H3K9ac enrichment in Populus trichocarpa under drought stress, which is presented in nine circles from 1 (innermost circle) to 9 (outermost circle). The inner-circle 1 represents gene density. Circle 2 shows H3K9ac enrichment distribution after 7 days drought. The color scale ranges from red (high enrichment) to blue (low enrichment). Circle 3 presents differential H3K9ac levels distribution under 7 days drought. The orange dot is increased both H3K9ac level and gene upregulation, the green dot represents a decrease in both H3K9ac level and gene downregulation, the blue dot shows opposite regulation of H3K9ac level and gene regulation, and the yellow dot shows only differential H3K9ac levels. Circle 4 presents differential genes after 7 days of drought (the orange dot shows increased H3K9ac level and gene upregulation, the green dot shows decreased in both H3K9ac level and gene downregulation, the blue dot shows opposite regulation of H3K9ac level and gene regulation, the yellow dot shows only differential gene regulation). Circle 5 presents H3K9ac enrichment distribution after 5 days of drought. Circle 6 shows differential H3K9ac levels distribution after 5 days of drought. Circle 7 presents differentially expressed genes after 5 days of drought. Circle 8 presents H3K9ac enrichment distribution after 0 days drought. Circle 9 presents chromosomes of P. trichocarpa and the red line in the track represents drought-responsive genes, which showed that H3K9ac modifications are enriched in drought-responsive genes. [file Image_1.pdf]

A

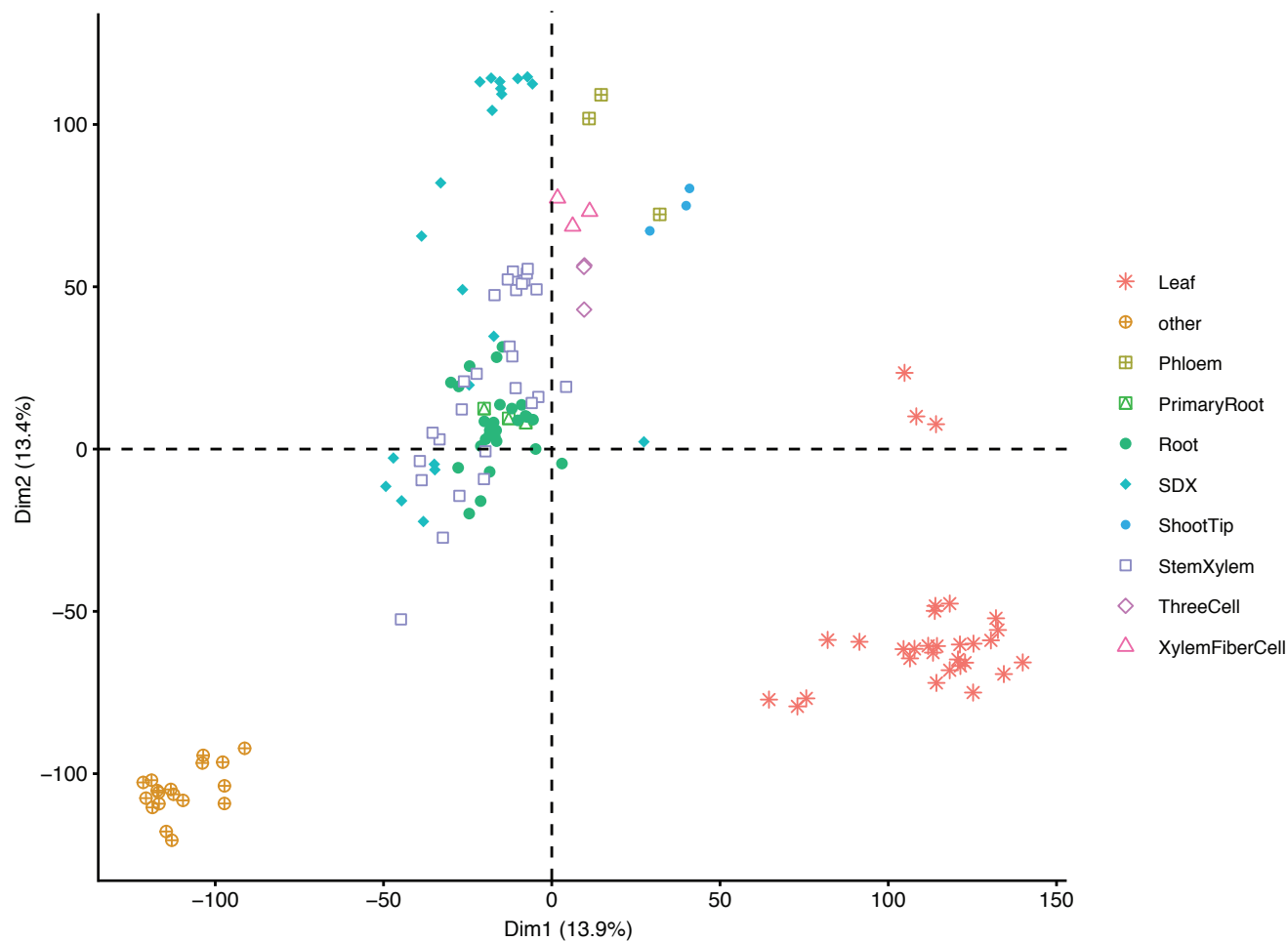

B

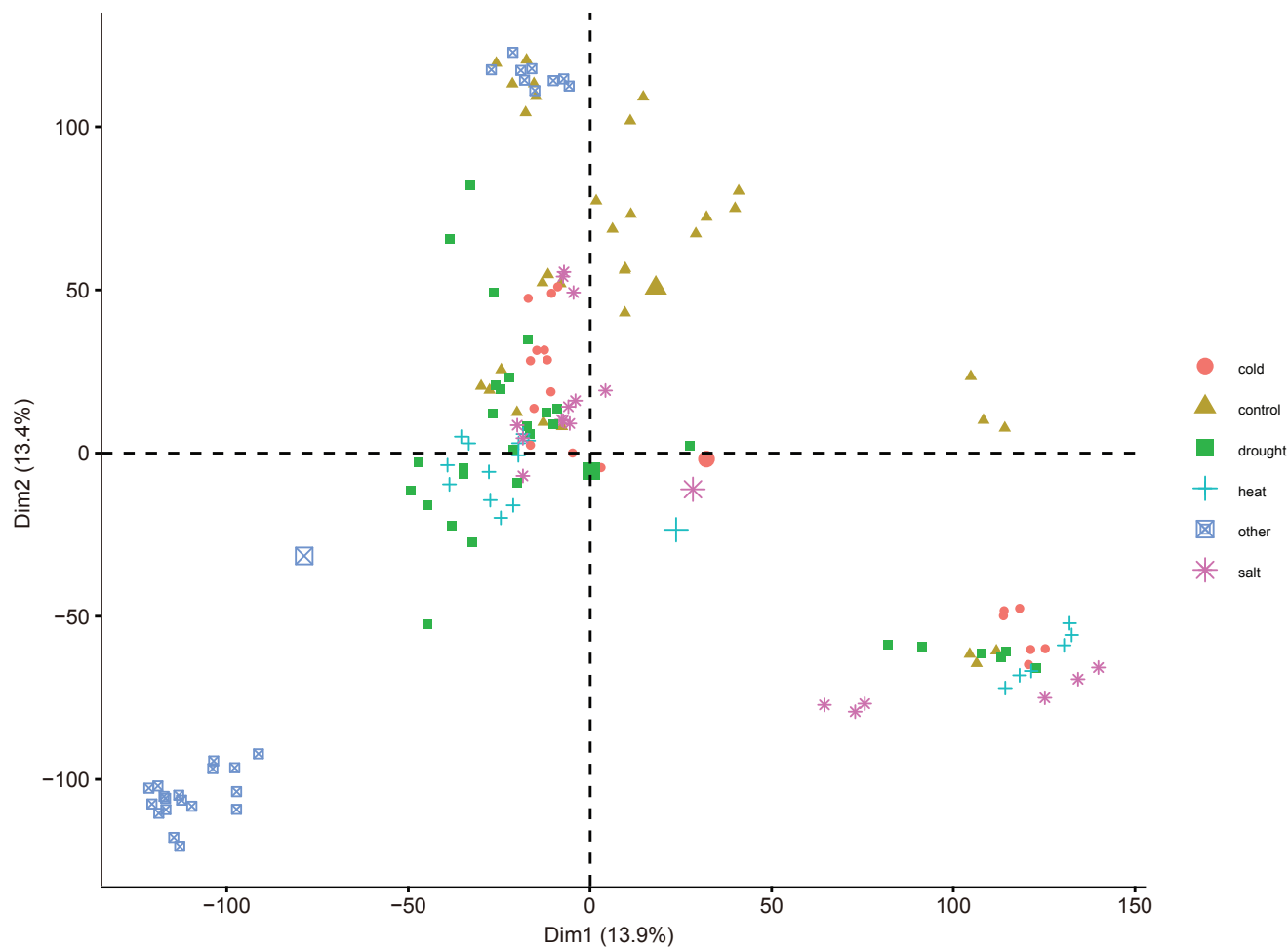

Supplement: Supplementary Figure 2 — Principal component (PC) analysis plots for different tissues and stress treatments. (A) Principal component (PC) analysis plots for different tissues. Different shape means different tissues. “Other” group represent protoplasts with miR397, SND1 or GFP overexpression. “SDX” group represent Stem Differentiating Xylem. (B) Principal component (PC) analysis plots for different stress treatments. Different shape means different treatments. “Other” group represent protoplasts with miR397, SND1, or GFP overexpression. [file Image_2.pdf]

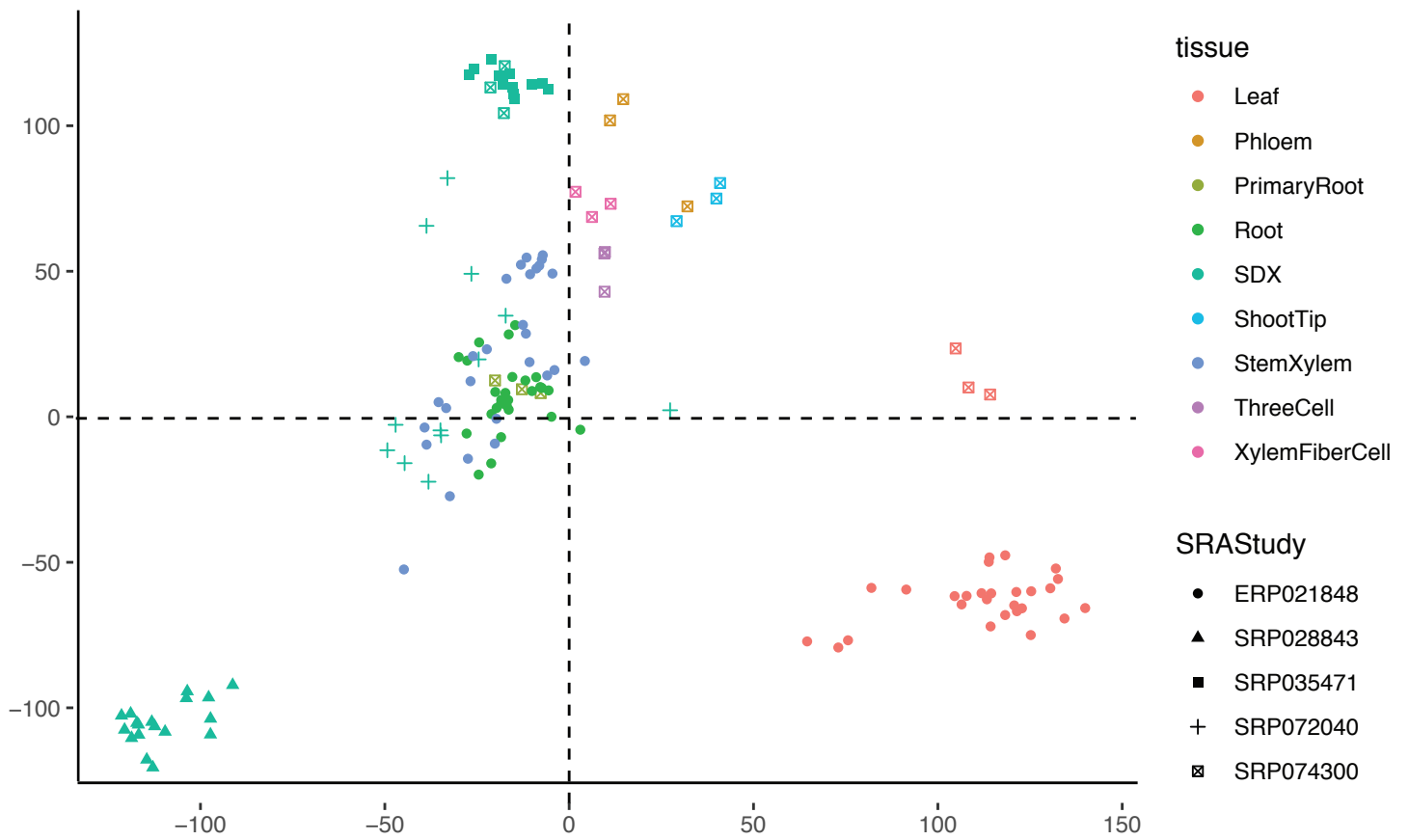

Supplement: Supplementary Figure 3 — Principal component (PC) analysis plots for different tissues and datasets. Different shape means different datasets. Different color means different tissue. “SDX” group represent Stem Differentiating Xylem. [file Image_3.pdf]

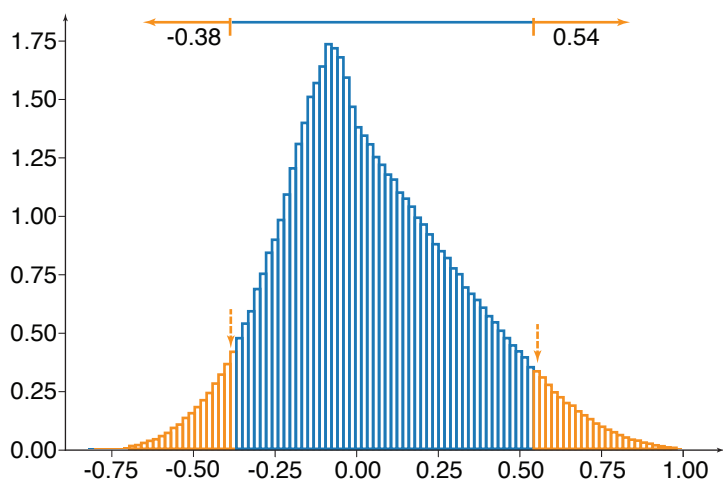

Supplement: Supplementary Figure 4 — The distribution of the Pearson Correlation Coefficient of stress-responsive genes under stress. [file Image_4.pdf]

A

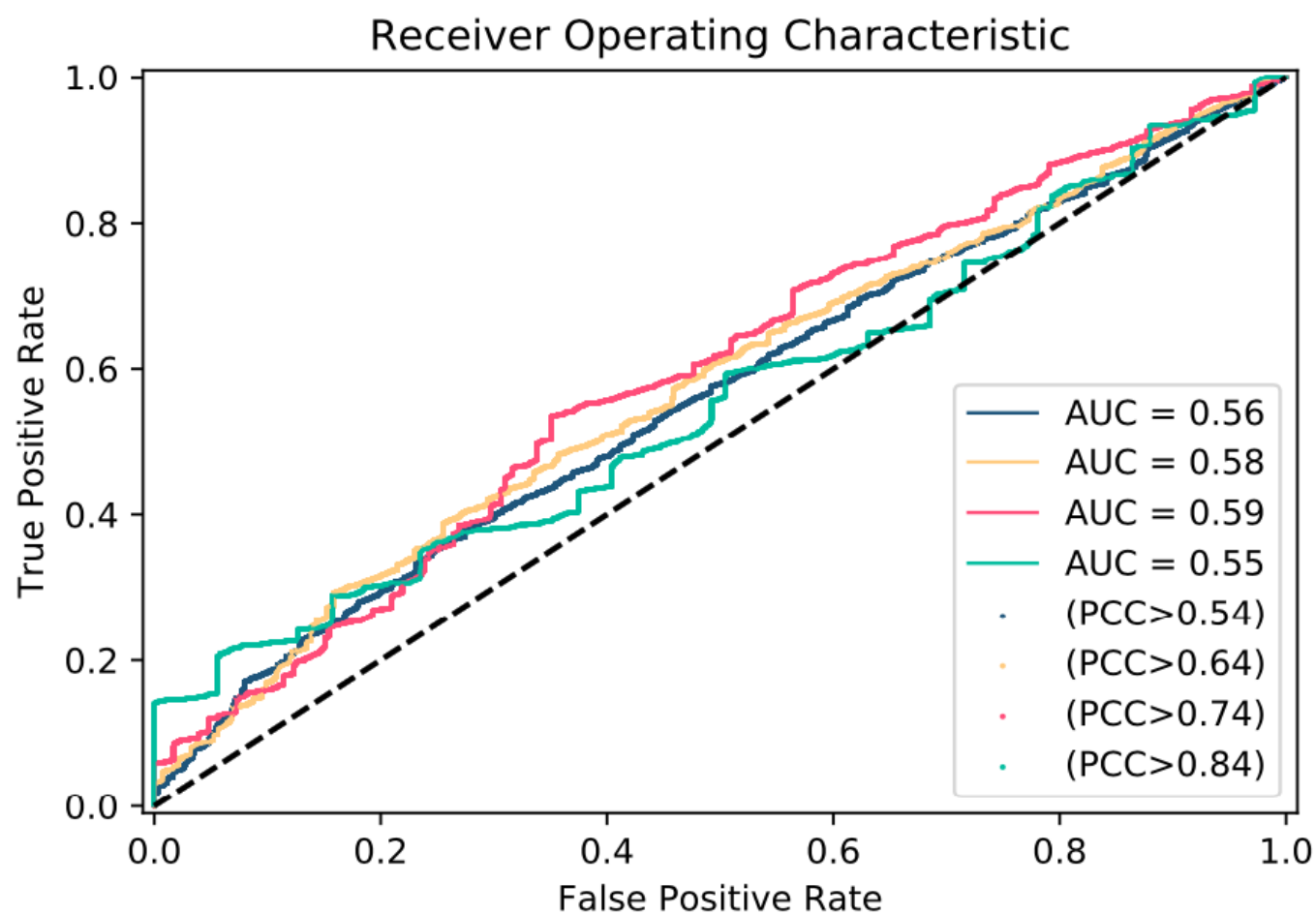

B

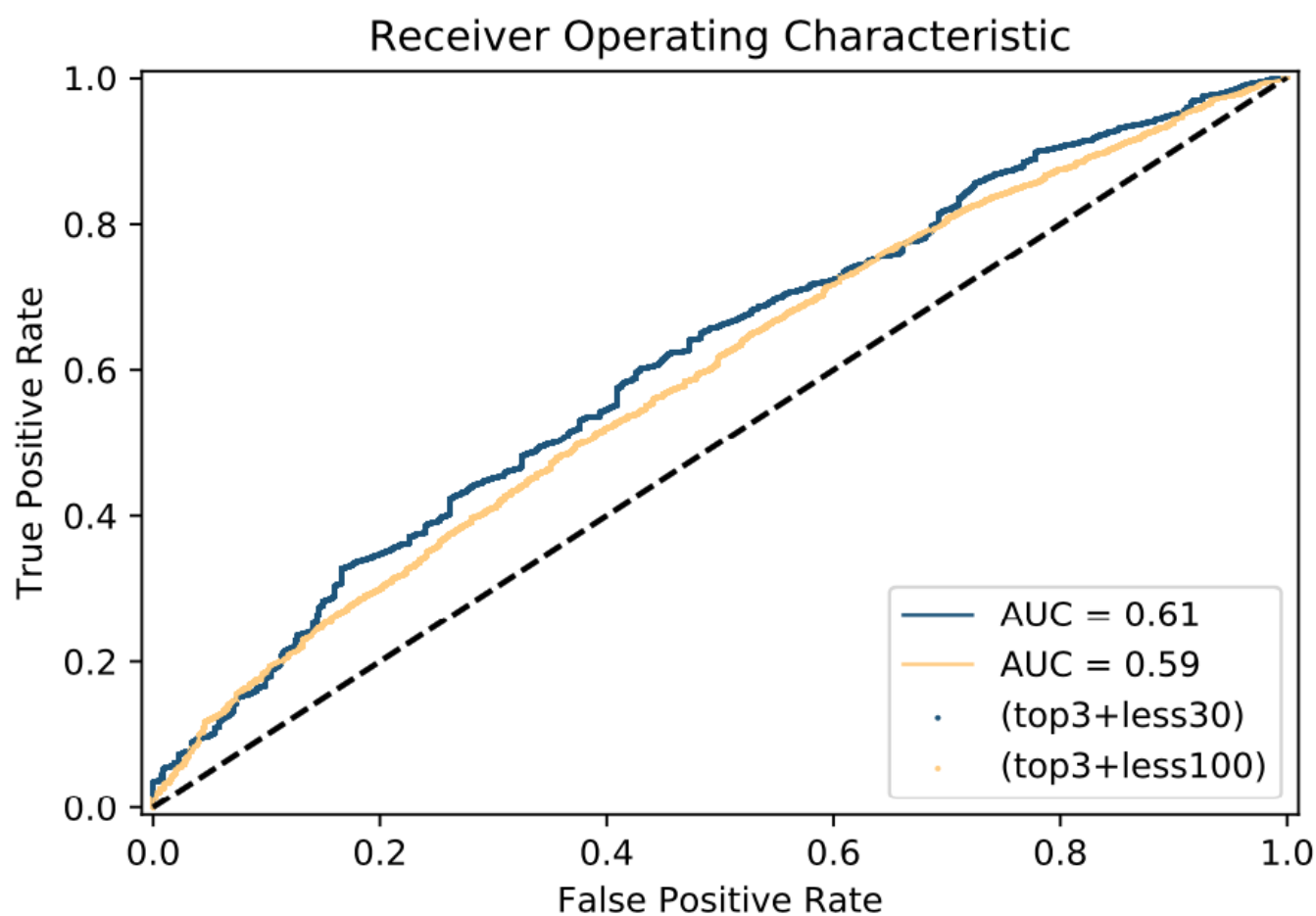

Supplement: Supplementary Figure 5 — MR values filtering for stress co-expression network. (A) Assessment of PCC co-expression network. (B) Assessment of MR co-expression network. [file Image_5.pdf]

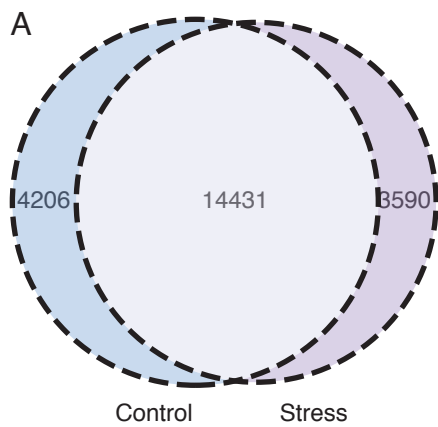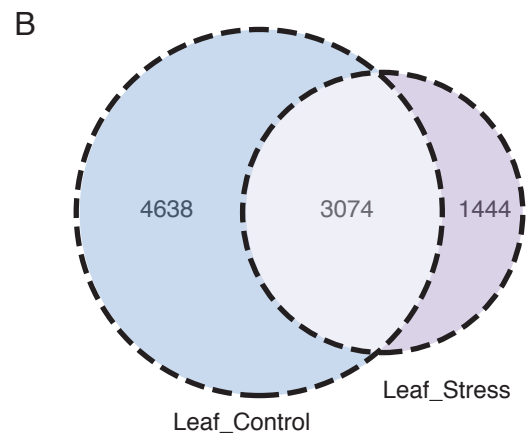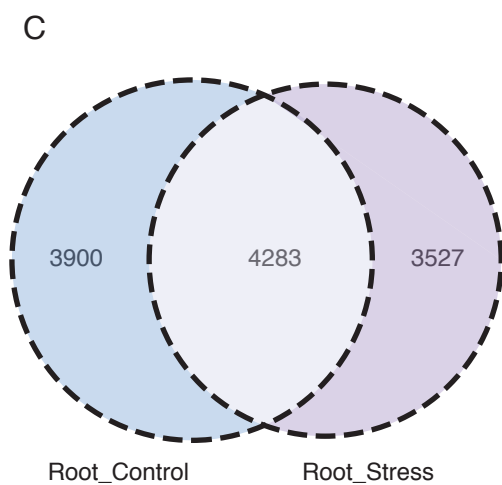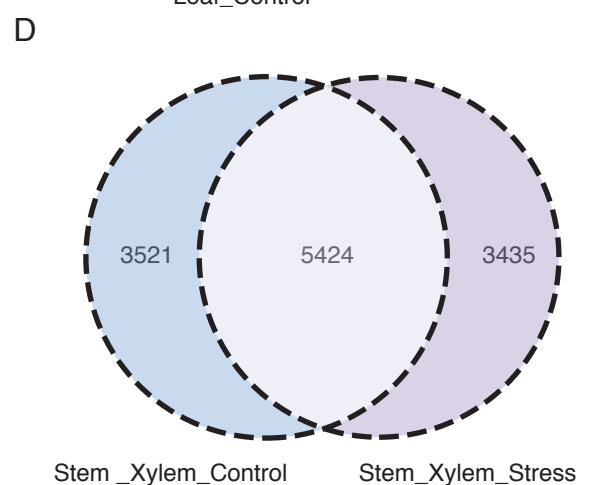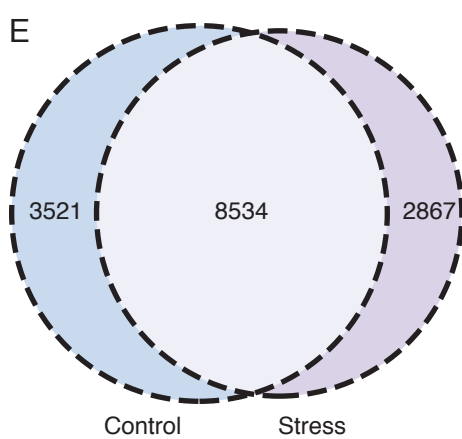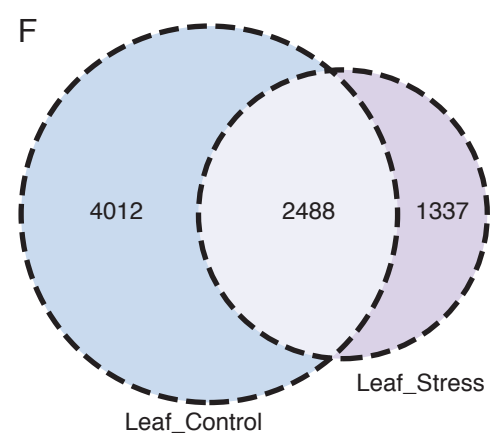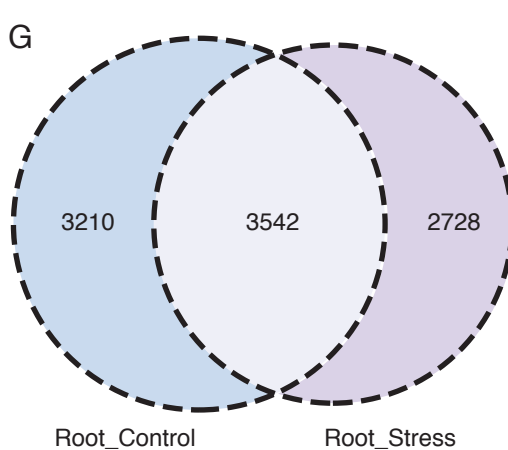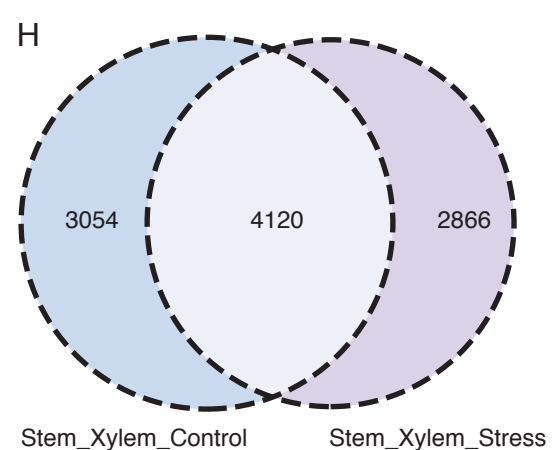

Supplement: Supplementary Figure 6 — The profile of alternative polyadenylation and alternative transcription initiation. (A) All APA genes in control and stress condition. (B) APA genes under stress and control in leaf. (C) APA genes under stress and control in root. (D) APA genes under stress and control in stem xylem. (E) All ATI genes in control and stress condition. (F–H) ATI genes in leaf, root and stem xylem compare control and stress. [file Image_6.pdf]

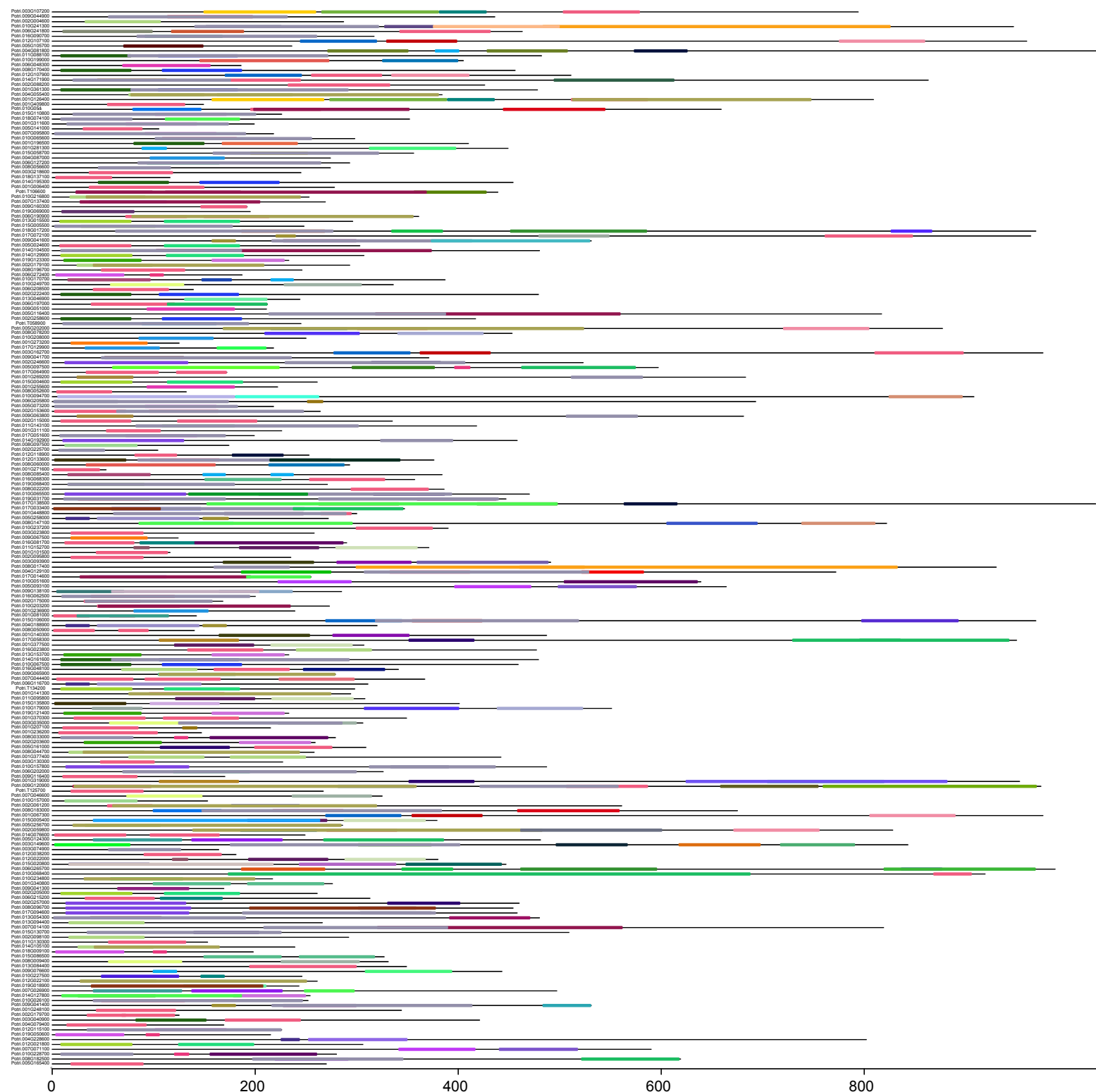

Supplement: Supplementary Figure 7 — Domain structure diagrams for RRM-domain protein. [file Image_7.pdf]
